# Dual-wavelength stopped-flow analysis of the lateral and longitudinal assembly kinetics of vimentin

**DOI:** 10.1016/j.bpj.2022.09.008

**Published:** 2022-09-13

**Authors:** Lovis Schween, Norbert Mücke, Stéphanie Portet, Wolfgang H. Goldmann, Harald Herrmann, Ben Fabry

**Affiliations:** 1 Department of Physics, Friedrich-Alexander-Universität Erlangen-Nürnberg, Erlangen, Germany; 2 Division of Chromatin Networks, German Cancer Research Center, Heidelberg, Germany; 3 Department of Mathematics, University of Manitoba, Winnipeg, Manitoba, Canada; 4 Institute of Neuropathology, University Hospital Erlangen, Erlangen, Germany; 5 Muscle Research Center Erlangen (MURCE), Friedrich-Alexander-Universität of Erlangen-Nürnberg, Erlangen, Germany

## Abstract

Vimentin is a highly charged intermediate filament protein that inherently forms extended dimeric coiled coils, which serve as the basic building blocks of intermediate filaments. Under low ionic strength conditions, vimentin filaments dissociate into uniform tetrameric complexes of two anti-parallel-oriented, half-staggered coiled-coil dimers. By addition of salt, vimentin tetramers spontaneously reassemble into filaments in a time-dependent process: 1) lateral assembly of tetramers into unit-length filaments, 2) longitudinal annealing of unit-length filaments, and 3) longitudinal assembly of filaments coupled with subsequent radial compaction. To independently determine the lateral and longitudinal assembly kinetics, we measure with a stopped-flow instrument the static light scattering signal at two different wavelengths (405 and 594 nm) with a temporal resolution of 3 ms and analyze the signals based on Rayleigh-Gans theory. This theory considers that the intensity of the scattered light depends not only on the molecular weight of the scattering object but also on its shape. This shape dependence is more pronounced at shorter wavelengths, allowing us to decompose the scattered light signal into its components arising from lateral and longitudinal filament assembly. We demonstrate that both the lateral and longitudinal filament assembly kinetics increase with salt concentration.

## Significance

The proper formation of intermediate filament networks in the cytoplasm is important for numerous cell functions. Here, we present a stopped-flow method for measuring the in vitro assembly kinetics of intermediate filaments with a temporal resolution of 3 ms using static light scattering at two different wavelengths. This allows us to compute the shape factor of the assembly products based on Rayleigh-Gans light scattering theory. From the shape factor, we can separately measure the lateral assembly of tetramers into unit-length filaments, as well as the longitudinal annealing of unit-length filaments and longer filaments. For the intermediate filament protein vimentin, we find that with increasing salt concentrations, both the lateral and longitudinal assembly rates increase, and unstable, hyper-aggregated assembly complexes emerge.

## Introduction

Intermediate filament (IF) proteins constitute a central structural element in metazoan cells. IFs form, together with the F-actin and microtubule system, cell-type-specific networks referred to as the cytoskeleton (1,2). IFs exhibit a very low persistence length in the range of 0.3–1.0 *μ*m for keratin and vimentin filaments, respectively (3,4). By contrast, the persistence length of F-actin is approximately 20 *μ*m (5), and the persistence length of microtubules is around 4–8 mm (6), depending on the presence of filament-stabilizing agents. IFs withstand standard disruptive treatments during isolation from tissues and are thus usually recovered from the insoluble fractions. IFs can then be solubilized into a monomeric form by treatment with chaotropic reagents such as urea. By resuspension in buffer of very low ionic strength, IFs dissolve into tetrameric complexes, as revealed by analytical ultracentrifugation (3,7, 8, 9, 10, 11, 12). By raising the ionic strength, tetramers spontaneously aggregate laterally in register and form so-called unit-length filaments (ULFs), which exhibit the same length as tetramers, i.e., ∼60 nm. Subsequently, ULFs anneal longitudinally into long filaments (Fig. S1). These reactions occur in the absence of bound nucleotides, contrary to the polymerization of actin and tubulin, which require that nucleotides are bound to the monomers. The formation of ULFs from tetramers via octamers and 16-mers is fast, with rate constants >100 *μ*M^−1^s^−1^. This process is largely completed within 1 s, as shown by light scattering measurements with a stopped-flow device (12).

Notably, mature ULFs contain 8, 10, or 12 tetramers per cross section (see Fig. S1). This indicates that the tetramers, through various permutations of transitional complexes such as octamers, 16-mers, and 24-mers, have assembled into higher-order complexes (12,13). After 60 min of assembly, however, long and comparatively uniform filaments of 8, 10, and 12 tetramers (38, 47, and 56 kDa/nm) per cross-section prevail (8,13). This indicates that the complexes formed in the very first phase of assembly are in a dynamic transition state with respect to the lateral packing of tetramers, eventually reaching a stable number of tetramers per cross section as larger, only weakly associated subcomplexes dissociate. This structural polymorphism appears not to be restricted to in vitro conditions, but it is also found in cells (8,14,15).

The aim of this experimental study was to gain deeper insights into the initial phase of filament elongation. Therefore, we have implemented a stopped-flow device equipped with two lasers of different wavelengths, 405 and 594 nm, for static light scattering measurements with a time resolution of 3 ms. By measuring the scattered light at two different wavelengths, we can distinguish between the lateral and longitudinal assembly kinetics of vimentin based on a simple principle: during the early phase of the assembly process when the tetramers are laterally aggregating into ULFs, the static light scattering signal at both wavelengths increases in proportion with the molecular weight of the reaction products. When filaments elongate by longitudinal annealing of ULFs, however, the scattered light increases less than proportionally with filament length, until no further significant increase in the scattered light intensity occurs. This is the case when filaments have grown beyond a length of ∼500 nm (11 ULFs) for incident light with a wavelength of 594 nm or ∼250 nm (5–6 ULFs) for incident light with a wavelength of 405 nm.

The wavelength and filament length dependence of the scattered light intensity can be described by the Rayleigh-Gans scattering theory. In particular, this theory allows us to compute the shape of the scattering objects and thus the longitudinal assembly rate from the ratio of the scattered light intensities at 594 and 405 nm. By contrast, lateral filament assembly equally affects the scattered light signals at different wavelengths and hence does not affect the intensity ratio. Thus, using a dual-wavelength stopped-flow approach, we can separately monitor the lateral and longitudinal components of the filament assembly process from the very beginning of the polymerization (3 ms) up to ∼10 min, when the filaments have reached a length of several ULFs.

Our data reveal that with increasing salt concentrations, both the lateral and longitudinal assembly rates of vimentin increase, and the mature filaments exhibit a higher mass per cross section. Intriguingly, we identify a salt and protein concentration-dependent transient reorganization phase (1–90 s after assembly start), during which distinctly larger complexes evolve that appear to be assembly incompetent and likely dissociate, as they are not found in mature filaments.

## Materials and methods

### Protein chemical methods

Human vimentin is expressed in transformed bacteria and *E. coli* strain TG1 and is isolated from inclusion bodies as described previously (10). For IF assembly, we use a Tris-HCl-buffered sodium chloride system. In brief, tetramers are re-natured by dialysis of monomers dissolved in 8 M urea with 10 mM Tris-HCl (pH 7.5) against a solution containing 5 mM Tris-HCl (pH 8.4) and 1 mM DTT. For the last dialysis step, the buffer is thoroughly degassed to prevent the formation of gas bubbles in the stopped-flow reaction chamber (4,10,12).

Assembly is initiated by addition of an equal volume of degassed salt buffer, i.e., low-salt buffer: 45 mM Tris-HCl (pH 7.0) with 100 mM NaCl; medium-salt buffer: 45 mM Tris-HCl (pH 7.0) with 200 mM NaCl; and high-salt buffer: 45 mM Tris-HCl (pH 7.0) with 320 mM NaCl. Hence, the ionic conditions in the assembly chamber are 1) low-salt buffer: 22.5 mM Tris-HCl (pH 7.5), 50 mM NaCl; 2) medium-salt buffer: 22.5 mM Tris-HCl (pH 7.5), 100 mM NaCl; and high-salt buffer 22.5 mM Tris-HCl (pH 7.5), 160 mM NaCl. Final protein concentrations for assembly are between 0.05 and 0.4 mg/mL (corresponding to 0.25–2 *μ*M).

### Atomic force microscopy

Atomic force microscopy (AFM) is performed using the tapping mode in air as described previously (4,12). Vimentin is assembled at 37°C in low-salt buffer at a protein concentration of 0.4 mg/mL. Assembly is stopped by 10-fold dilution with low-salt buffer followed by fixation with an equal volume of 0.2% glutaraldehyde in low-salt buffer for 1 min. 40 *μ*L are then deposited on freshly cleaved mica. After 1 min, the mica samples are washed with double-distilled water and dried with a steady stream of nitrogen.

### Transmission electron microscopy (TEM)

Samples for visualization by TEM are fixed with freshly prepared 0.2% glutaraldehyde in assembly buffer. The filament suspension is briefly adsorbed to glow-discharged carbon-coated electron microscopic grids, washed with distilled water, and negatively stained with 2% uranyl acetate for 15 s (10). Specimens are examined in a Zeiss (model 910, Carl Zeiss, Oberkochen, Germany) transmission electron microscope.

The filament width is determined near the center of the filaments using the image analysis software Clickpoints (16). The diameters of at least 77 filaments for each time point (2, 10, 30, 60, 180, and 300 s after assembly start) are measured.

### Immunofluorescence microscopy

The filament suspension is absorbed on ethanol-cleaned glass slides for 1 min followed by fixation in methanol (6 min) and acetone (30 s) at −20°C. Primary rabbit anti-vimentin antibodies (17) and fluorescently labeled secondary antibodies (Jackson Laboratories, Bar Harbor, ME, USA) are used to visualize the fixed filaments (18).

### Stopped-flow experiments and data management

**Figure 1:**
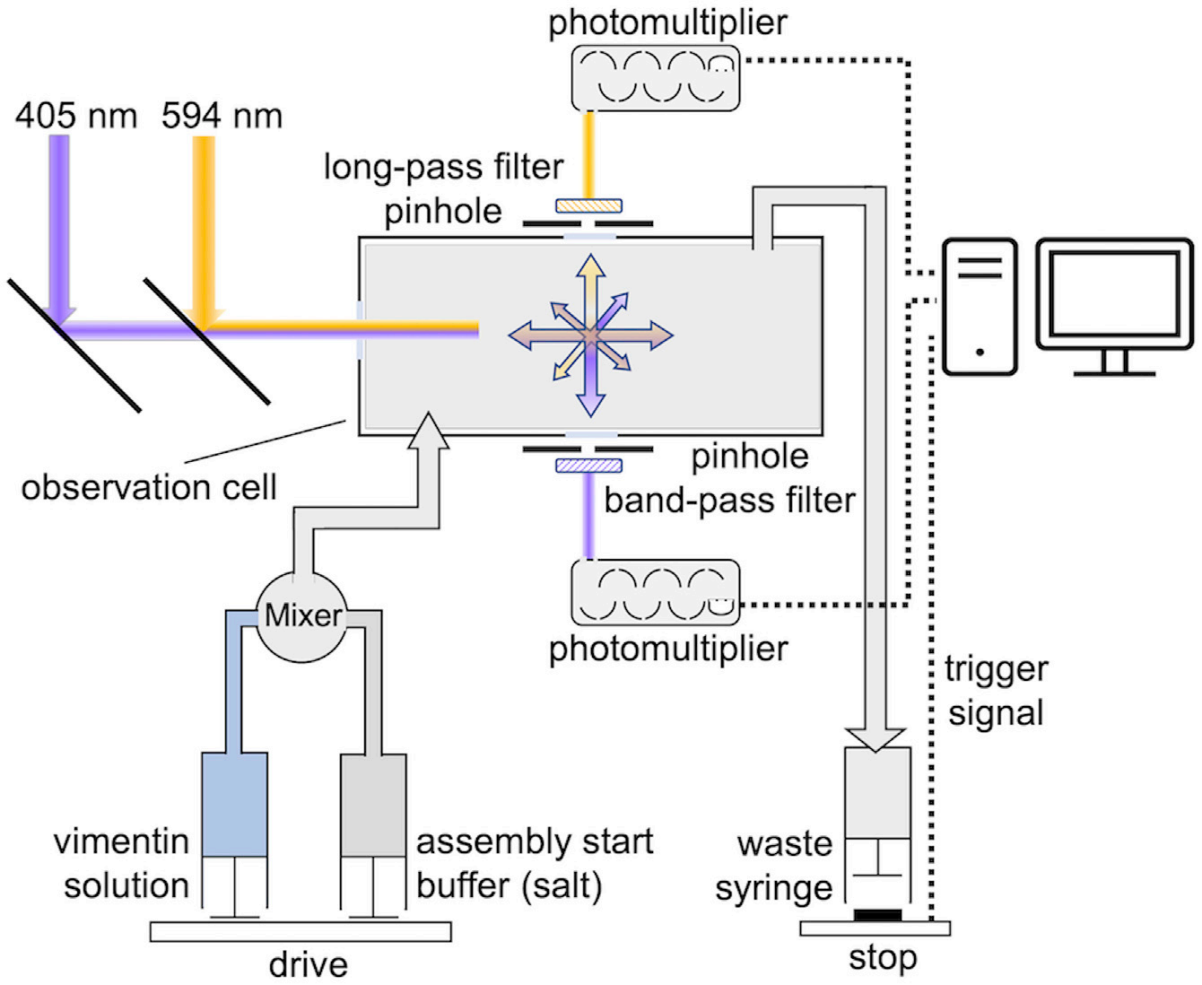
Schematic of the dual-laser stopped-flow setup. The aligned 405 and 594 nm laser beams are guided into the center of the observation chamber. Perpendicular to the incident beam, the scattered light for both wavelengths is led through a pinhole and a band-pass (405 nm) and a long-pass (594 nm) filter and detected with a photomultiplier. To see this figure in color, go online.

Stopped-flow measurements are performed with a two-syringe stopped-flow apparatus (SF-61, Hi-Tech Scientific, Salisbury, UK) as described in detail in (12) (Fig. 1). In brief, one syringe is filled with soluble vimentin protein (concentration between 0.1 and 0.8 mg/mL) and the other syringe with the assembly buffer containing 45 mM Tris-HCl and 100–320 mM NaCl (pH 7.0). All solutions are filtered through 0.22 *μ*L syringe filters (Rotilabo; Carl Roth, Karlsruhe, Germany). Both syringes are heated to 37°C and allowed to equilibrate for 5 min before starting the measurements.

For measurements, both syringes are emptied simultaneously by a drive platform powered by air pressure at 400 kPa. Equal volumes from both syringes are rapidly (∼3 ms) mixed and driven into the detection chamber, displacing the solution from the previous run, which is discarded into the waste syringe. The flow rate of the solution in the system under these conditions is ∼1.5 mL/s. The flow is stopped when the plunger of the waste syringe hits the stopping block after 200 *μ*L, triggering the data acquisition system. The dead time (delay between trigger activation and signal increase) is 3 ms (12).

Prior to assembly experiments, both syringes are rinsed with several milliliters of degassed ddH2O until no change in the light scatter signal is detected. This signal then serves as a baseline for further measurements. In a second step, both syringes are rinsed with several milliliters of tetramer buffer until no change in the light scatter signal is detected. This signal is used to calibrate the light scatter signal. Static light scatter is measured at wavelengths of 594 and 405 nm delivered by diode-pumped solid-state lasers (405 nm: Flexpoint 25 mW; Lasercomponents, Olching, Germany; 594 nm: Mambo 25 mW; Cobolt, Solna, Sweden). The light is transmitted via a flexible light guide to the observation chamber. The scattered light is band-pass filtered (405 nm) and long-pass filtered (594 nm) (Schott AG, Mainz, Germany) and detected by two photomultipliers (PM-60s, Hi-Tech Scientific, Salisbury, UK). The electric photomultiplier signals are low-pass filtered (time constant: 1 ms). Differential signals are digitized (PCI-6023E, National Instruments, Austin, TX, USA) at a resolution of 12 bit and sampled at 1 kHz. Measurements that exhibit large fluctuations caused by air bubbles are discarded.

### Shape factor calculation

For a solution of randomly oriented rod-like particles such as IFs with a length > *λ*/10, when *λ* is the wavelength of the incident light, and a width < *λ*/10, Rayleigh-Gans scattering theory considers that the scattered light depends not only on the molecular weight of the scattering object (as in the case of simple Rayleigh scattering) but also on its shape (19). Accordingly, the scattered light intensity *I* at an angle *θ* between the incoming and scattered light direction can be expressed as the scattered light intensity $I_{0}$ that would be expected for simple Rayleigh scattering, times a shape factor P(θ):

(1) $$I=I_{0}P\left(\theta \right).$$

In our setup, the angle *θ* = 90°. In this case, the shape factor for randomly oriented rod-shaped cylindrical particles with length *l* and radius *r* can be computed as the product of a radius-dependent function F(r) and a length-dependent function E(l),

(2) $$P\left(90^{\circ }\right)=F{\left(r\right)}^{2}E{\left(l\right)}^{2}.$$

For radii <10 nm, as is the case for IFs, the radius-dependent function F(r) tends to unity. The length-dependent function E(l) depends on the wavelength *λ* and can be expressed as

(3) $$E^{2}\left(z\right)=\frac{1}{z}\int_{0}^{2z}\frac{\text{sin}\omega }{\omega }\text{d}\omega -{\left(\frac{\text{sin}z}{z}\right)}^{2},$$

with

(4) $$z=\frac{2\pi l}{\lambda }\text{sin}\frac{\pi }{4}.$$

We assume that a single vimentin ULF has a length of 60 nm and that the length increase due to partial filament overlap is 43 nm for each additional ULF (20,21).

**Figure 2:**
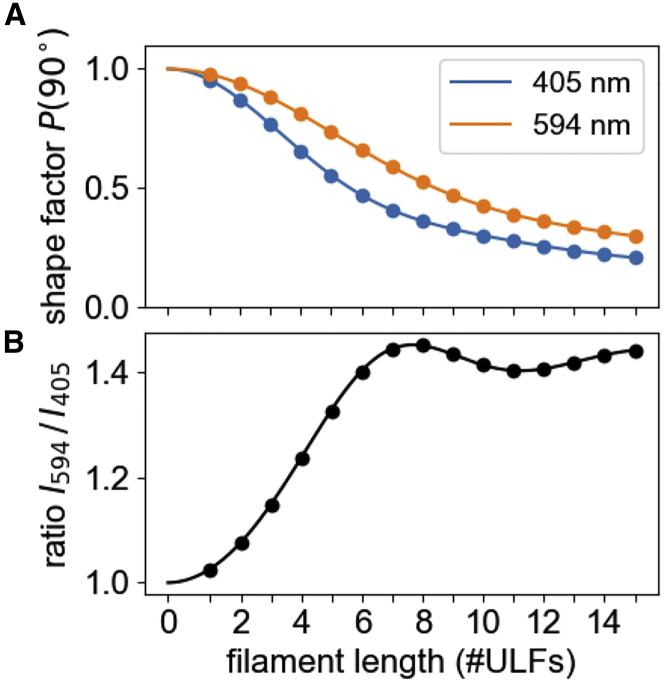
(*A*) Shape factor for different wavelengths for a scattering angle of 90° versus filament length as predicted by Rayleigh-Gans theory (Eqs (2), (3), (4)). The shape factor determines the normalized scattered light intensities for different filament lengths. (*B*) The ratio of the scattered light intensities (594/405 nm) increases monotonically during filament elongation up to a filament length of 7 ULFs. To see this figure in color, go online.

Because the shape factor is wavelength dependent (Fig. 2 *A*), we can estimate the shape factor, and hence the average length of the scattering particles, from the ratio of the scattered light intensity measured at different wavelengths (Fig. 2 *B*) if two conditions are met: the shape of the filament length distribution must be known at least approximately (it is log normal in the case of vimentin), and the average filament length must remain below ∼6–7 ULFs, beyond which the scattered light intensity ratio shows a local maximum (Fig. 2 *B*) and little further changes. Importantly, however, the scattered light intensity ratio is largely insensitive to any lateral filament assembly and heterogeneity in thickness, as radial size differences equally affect the scattering signal at different wavelengths.

## Results

### Longitudinal filament assembly measured by AFM

**Figure 3:**
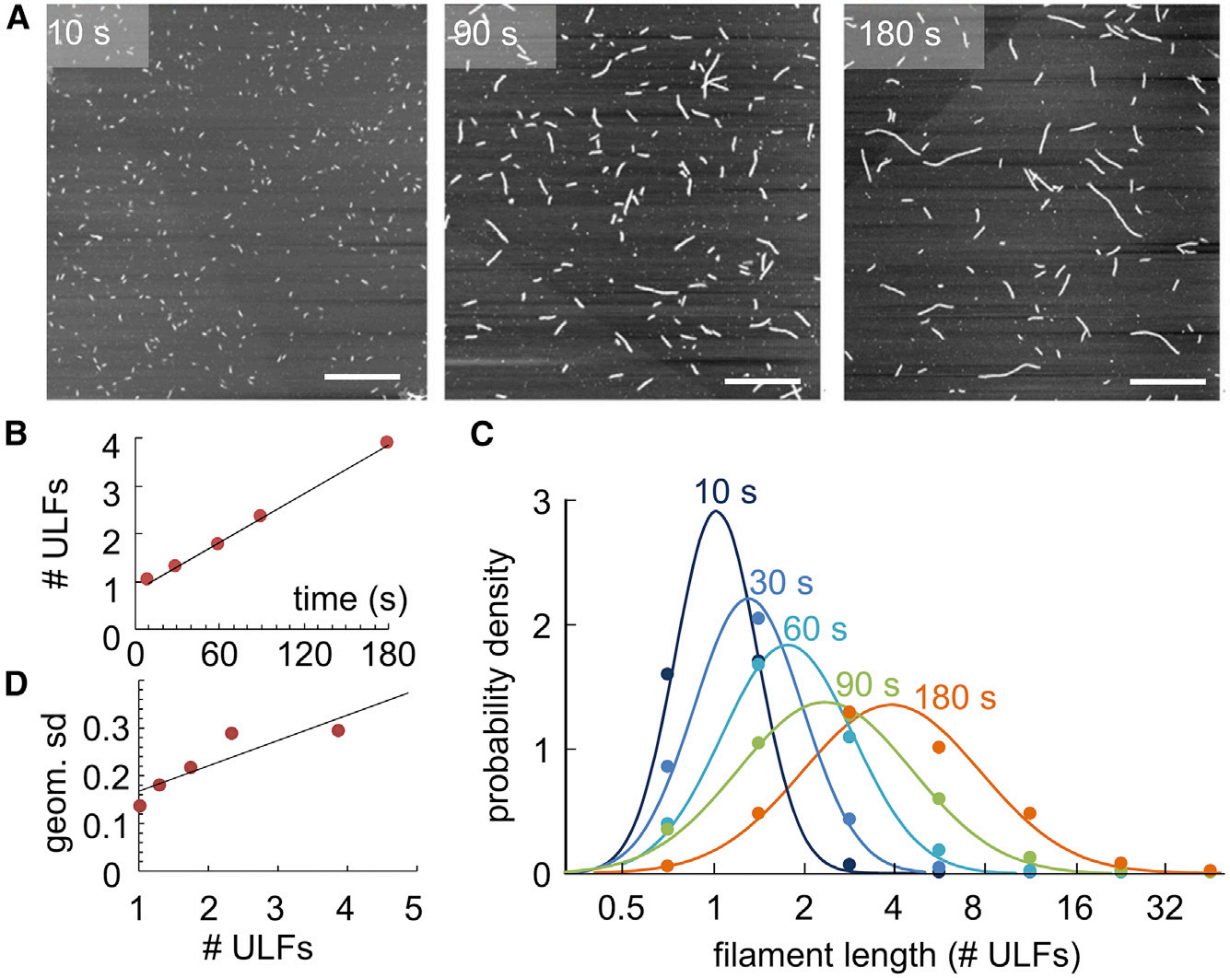
Vimentin elongation measured with AFM. (*A*) Vimentin filaments imaged with AFM after 10, 90, and 180 s after assembly start. Scale bar is 1 *μ*m. (*B*) Geometric mean of filament length versus assembly time. The black line indicates a linear fit to the data, with a slope of 1.02 ULFs/min. (*C*) Distribution of filament lengths at different time points after starting assembly. Points indicate measurements of probability density from AFM images in logarithmically spaced bins (0.5–1 ULF, 1–2 ULFs, 2–4 ULFs, etc.); lines are a log-normal fit to the data. (*D*) Geometric standard deviation of filament length distribution versus geometric mean filament length. The black line indicates a linear fit to the data, with $\sigma =0.053l_{fil}+0.115$, when the filament length $l_{fil}$ is given in units of #ULFs. To see this figure in color, go online.

Vimentin at a concentration of 0.4 mg/mL is assembled in low-salt buffer (50 mM NaCl in 25 mM Tris-HCl [pH 7.5]), and the assembly reaction is stopped by a 10-fold dilution with low-salt buffer, followed by fixation by addition of an equal volume of 0.2% glutaraldehyde dissolved in low-salt buffer. The filaments are then adsorbed on mica, washed, and dried under a steady stream of nitrogen. AFM measurements (Fig. 3 *A*) confirm a linear increase of filament length with time (Fig. 3 *B*). We find a longitudinal assembly rate of 1.02 ULFs/min. At each time point, the lengths of the individual filaments show an approximately log-normal distribution (Fig. 3 *C*). The width of the distribution increases monotonically with increasing assembly time and hence with increasing average filament length $l_{\text{fil}}$. In particular, we find that the geometric standard deviation of the filament length distribution, *σ*, increases monotonically with increasing average filament length (Fig. 3 *D*). This relationship can be empirically expressed with a linear relationship

(5) $$\sigma =0.053\;l_{fil}+0.115,$$

where $l_{fil}$ is the geometric mean filament length expressed in units of #ULFs. We have verified, using published data, that a linear increase of the geometric standard deviation with increasing average filament length can also be observed for assembly of vimentin in KCl salt. These previous measurements indicate that the slope of the relationship may depend on the salt and vimentin concentration, but the intercept remains constant, with values around 0.12.

### Lateral assembly measured by electron microscopy (EM)

**Figure 4:**
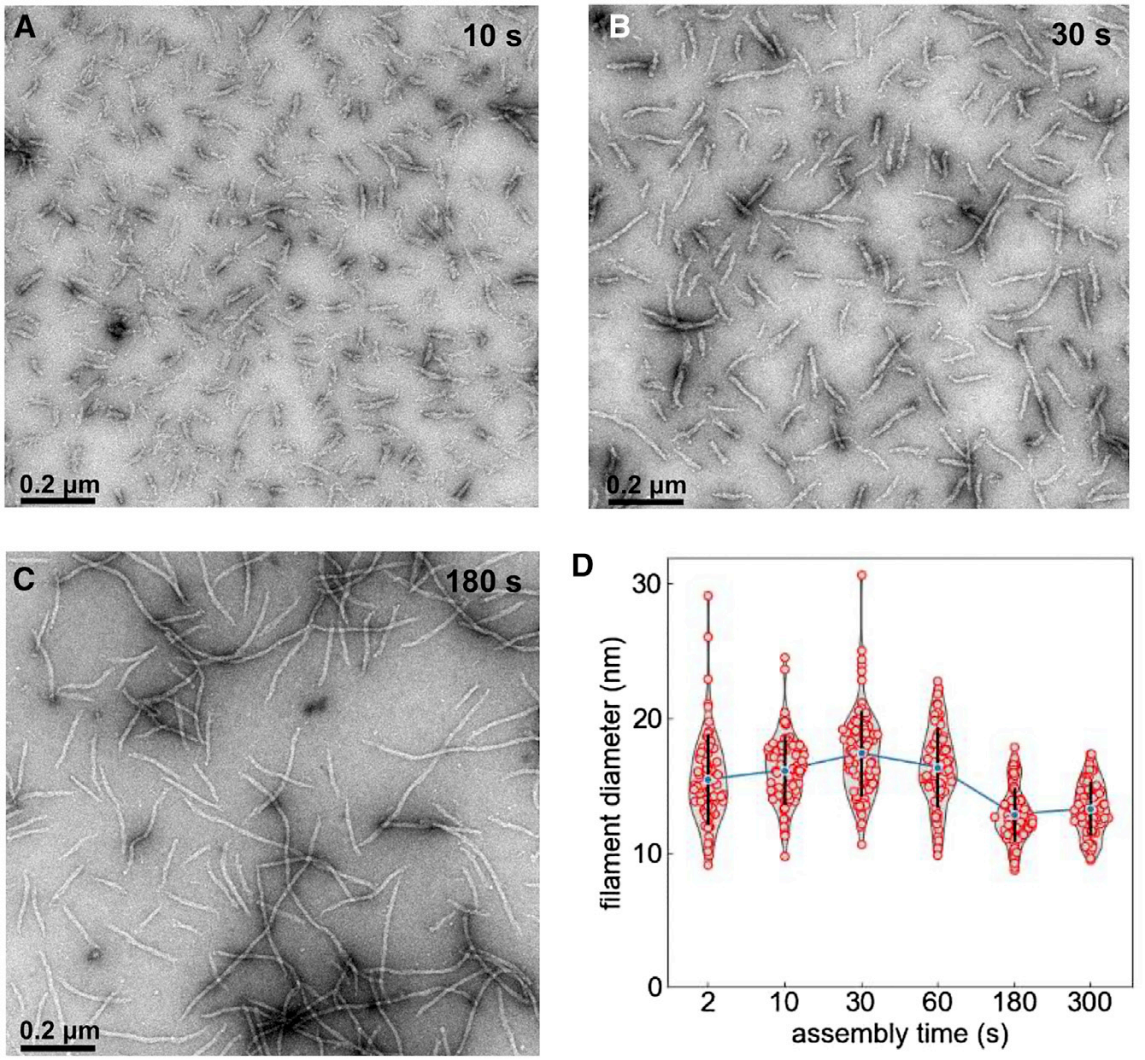
(*A*–*C*) Assembly of vimentin filaments (0.2 mg/mL in 50 mM NaCl) at 37°C for 10 (*A*), 30 (*B*), and 180 s (*C*). Transmission electron microscopy of glutaraldehyde-fixed and uranyl acetate negatively stained specimens. Scale bar: 200 nm. (*D*) Filament diameter and distribution at various time points. Red points represent individual measurements, blue points show the average, and black bars denote standard deviation. To see this figure in color, go online.

We use TEM images of vimentin filaments (0.2 mg/mL in 50 mM NaCl), fixated at 2, 10, 30, 60, 180, and 300 s after starting assembly, to measure the filament diameter, which reflects the number and density of tetramers that form the ULFs (Fig. 4 *A*–*C*). We find that the average filament diameter increases during the first 30 s of assembly from 15.5 to 17.5 nm and then decreases to 13 nm during the following 150 s, after which time point the average diameter remains approximately constant (Fig. 4 *D*). This diameter agrees with a previous study (13 nm after 5 min, 12 nm after 10 min, 11 nm after 60 min), where the salt concentration for assembly was 100 mM NaCl (22).

Interestingly, the distribution of filament diameters decreases over this time period. While we find some very thick filaments with diameters of up to 30 nm during the first 30 s after the start of assembly, all filaments (from 150 measurements) are thinner than 18 nm after 180 s. The mean filament diameter at 30 s of assembly is 30% larger than at 180 and 300 s. We interpret the reduction in both the mean and the distribution of the filament diameter as the result of an ongoing compaction process, including intrafilamentous reorganization and dissociation reactions of fragments from large ULFs (13).

### Longitudinal assembly measured with a dual-wavelength stopped-flow device

We can separate the scattered light signal resulting from lateral versus longitudinal assembly by applying Rayleigh-Gans theory. This theory considers the wavelength-dependent decrease of the scattered light signal for elongated scatterers relative to the prediction from Rayleigh theory, which assumes point-like scatterers (Fig. 2 *A*). According to Rayleigh-Gans theory, the shape factor for shorter wavelengths decreases more rapidly with increasing filament length compared with longer wavelengths.

We take advantage of this wavelength dependency by computing the ratio $r_{I594/I405}$ of the scattered light intensities at 594 nm relative to 405 nm. This ratio only depends on the shape factor and, hence, on the filament length. Once the filaments have reached a length larger than the wavelength of the incident light, the scattered light signal no longer increases as more ULFs are added, and the ratio $r_{I594/I405}$ asymptotically approaches a value of 1.46.

If filament length grows linearly with time, as seen in our data (Fig. 3 *B*), and if all filaments have a uniform length, the ratio $I_{594}/I_{405}$ nm of the scattered light intensities, when plotted versus time *t*, would exactly follow the ratio of the shape factors as shown in Fig. 2 *B*. The effect of log-normal-distributed filament lengths, however, blurs the time course of the ratio $r_{I594/I405}$ so that it resembles an exponential function

(6) $$r_{I594/I405}\left(t\right)=1+0.46\left(1-e^{-t/\tau }\right),$$

with time constant *τ* as a free parameter. The prefactor 0.46 corresponds to the asymptotic value of the shape factor ratios for infinitely long filaments $P{\left(90^{\circ }\right)}_{594}/P{\left(90^{\circ }\right)}_{405}$, minus unity. In practice, we allow this prefactor also to be a free fit parameter, as the experimentally obtained prefactors can range between values of 0.3–0.5. Fit values for the prefactor that are larger than 0.5 indicate in most cases that the measurement was too short to reliably fit a time constant.

**Figure 5:**
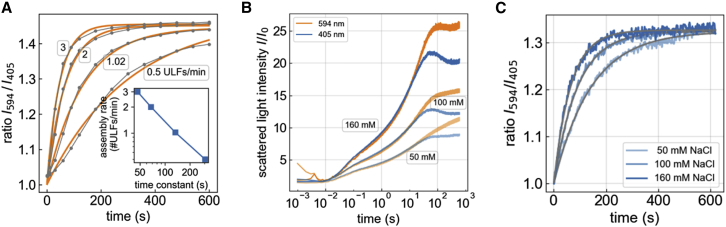
(*A*) Monte Carlo simulation of the ratio of the scattered light intensities at 594 and 405 nm for different longitudinal assembly rates and time points after starting the assembly (*gray curves*). The filament length and its distribution versus time is assumed to follow the relationship shown in Figs. 2*B* and 3*D*. The orange lines represent the fit of an exponential function (Eq. 6) to the data. The time constant *τ* of the exponential fit scales inversely with the longitudinal assembly rate $r_{la}$, according to $r_{la}$= 2.32 ULFs/*τ*, if *τ* is given in units of min (*inset*). (*B*) Scattered light intensities at 594 (*orange*) and 405 nm (*blue*) over time for assembly of 0.2 mg/mL vimentin in low-, medium-, and high-salt buffer. The scattered light intensities at 405 and 594 nm develop synchronously but begin to diverge beyond 10 s due to the wavelength-dependent scattering properties of elongated filaments. Representative measurements for all other conditions are shown in Fig. S3. (*C*) Scattered light intensity ratios (594/405 nm) for 0.2 mg/mL vimentin (*blue*) measured in low-, medium-, and high-salt buffer, with exponential fits (Eq. 6; *gray*). Representative curves for all other conditions are shown in Fig. S3. To see this figure in color, go online.

Although Eq. 6 provides only an empirical description, it closely describes both a computer-simulated and experimentally measured time evolution of the ratio $r_{I594/I405}$ (Fig. 5 *A* and *C*). The time constant *τ* of the fit scales inversely with the longitudinal assembly rate $r_{la}$ (see Fig. 5 *A* inset) according to

(7) $$r_{la}=\frac{2.32}{\tau },$$

where $r_{la}$ is given in units of ULFs/min if *τ* is given in units of min. The factor of 2.32 is determined under the assumption that the relationship between filament length and length distribution that we experimentally determined for 0.4 mg/mL vimentin in low-salt (50 mM NaCl) conditions (Fig. 3 *D*) also holds for other conditions, which we have not independently verified. Nonetheless, regardless of the specific value for the length distribution, Eq. 6 is still applicable, and the time constant *τ* of the fit scales inversely with the longitudinal assembly rate.

**Figure 6:**
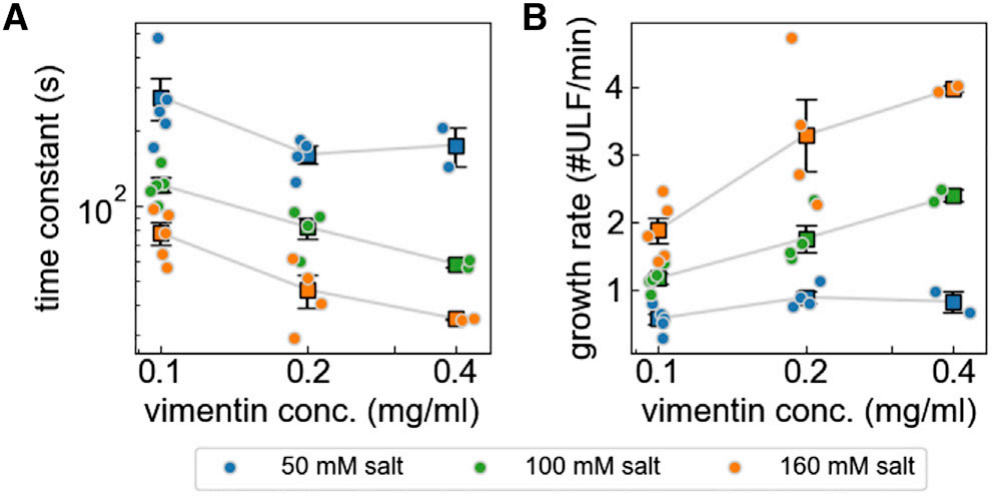
(*A*) Time constants of exponential fits (Eq. 6) to the measured 594/405 nm ratios (mean ± SE in *black*, individual measurements in *color*) for different vimentin and salt concentrations. (*B*) Estimated rate of filament elongation (assembly rate $r_{la}$) based on Eq. 7. To see this figure in color, go online.

We find that the longitudinal assembly of vimentin depends on both the vimentin and the salt concentration (Fig. 6). For a fourfold increase in vimentin concentration (from 0.1 to 0.4 mg/mL), we see approximately a doubling of the filament growth rate under medium- and high-salt conditions, specifically from 1 to 2 ULFs/min for medium salt and from 2 to 4 ULFs/min for high salt. By contrast, under low-salt conditions, the filament growth rate increases only slightly with increasing vimentin concentration.

### Lateral assembly measured with a dual-wavelength stopped-flow device

The time course of the scattered light intensities (Fig. 5 *B*) can be roughly subdivided into three distinct phases: 1) from 10 ms to 1 s after starting the reaction, the scattered light signal is dominated by the lateral assembly of tetramers into ULFs. Longer filaments have not yet formed, so that the signals at 405 and 594 nm appear identical. The signal intensity rises quickly at first but then, after a first inflection point at around 100 ms, more slowly, without reaching a plateau. Both the speed and the magnitude of the signal intensities increase with increasing salt concentrations. 2) At around 1–5 s, the scattered light signal shows a second inflection and picks up speed, indicating that longitudinal assembly has started to contribute to the scattered light signal. At around 5–30 s, depending on salt concentration, a fraction of the filaments has grown to a length of 2 ULFs or longer, and thus the 405 nm signal falls below the 594 nm signal. 3) The 405 nm signal shows a third inflection point at around 30–60 s (and in the case of medium- and high-salt concentrations, the signals even decrease) before leveling out toward the end of the recording at 600 s. Since the filaments still continue to grow in length, this signal decrease indicates that the filaments undergo a radial compaction. The 594 nm signal, which is less affected by the highly elongated shape of the filaments, shows its third inflection point later at around 100 s and then decreases only slightly and only at the highest salt conditions before leveling out. The signal plateau indicates that the filaments have reached a length beyond which the scattering signal becomes insensitive to any further growth. The signal plateau, moreover, is considerably higher for higher salt concentrations, indicating that the filaments have a larger mass per cross section.

**Figure 7:**
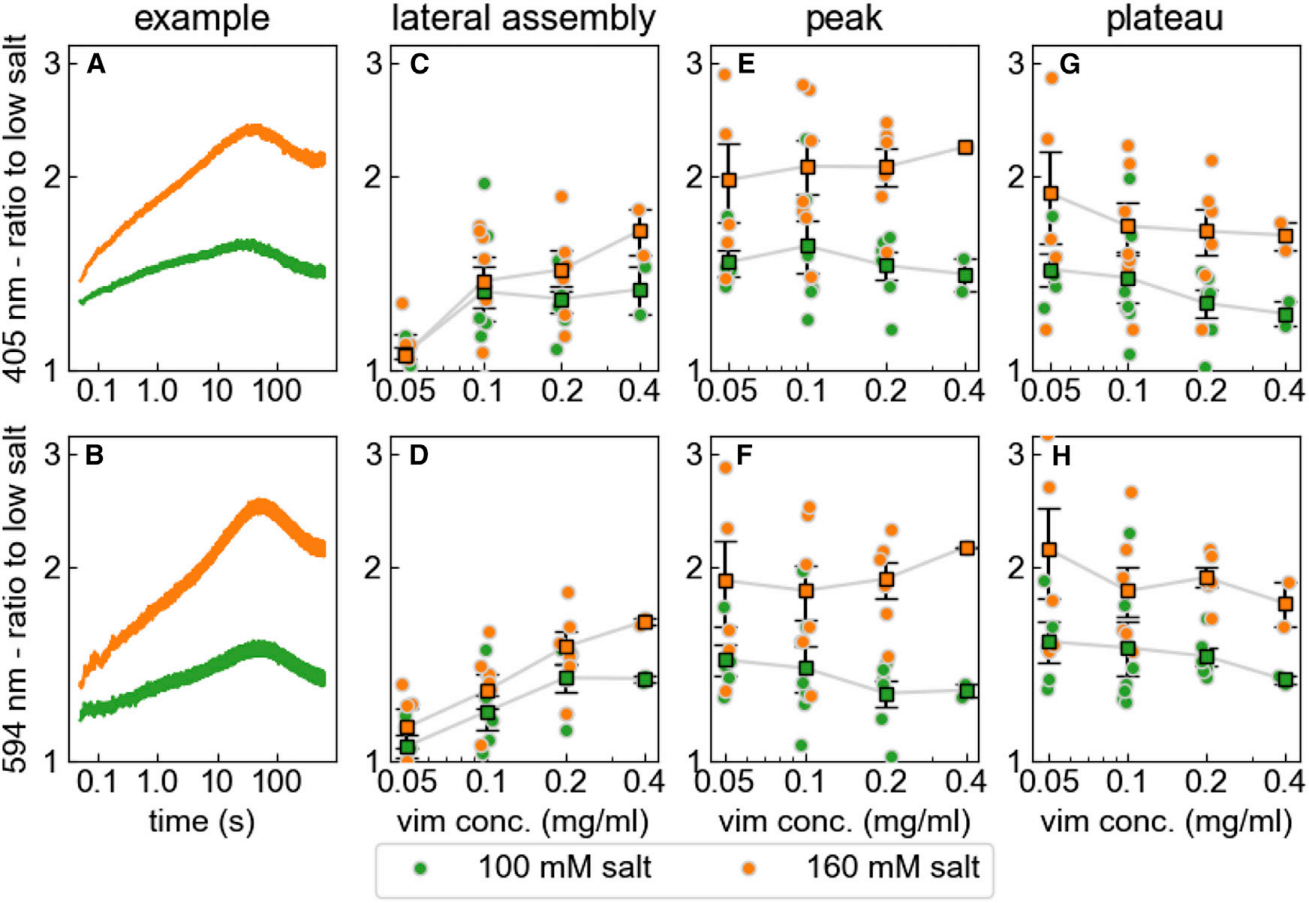
(*A* and *B*) Light scattering signal at 405 (*top row*) and 594 nm (*bottom row*) during assembly of 0.2 mg/mL vimentin in 100 mM (*green*) and 160 (*orange*) salt, normalized by the signal measured for 50 mM salt. (*C* and *D*) Signal ratio (*green*: 100 mM/50 mM; *orange*: 160 mM/50 mM) as shown in (*A*) and (*B*) averaged between 20 and 40 ms after assembly start, reporting differences in the lateral assembly kinetics relative to the assembly at low-salt conditions. (*E* and *F*) Ratio averaged over a 40 s wide interval around the intensity peak, combining contributions from lateral and longitudinal assembly. (*G* and *H*) Ratio averaged between 500 and 600 s after assembly start, reporting differences in the mass per cross section of the filaments relative to filaments assembled in 50 mM salt buffer. *Square* symbols show mean ± SE, *circles* show individual measurements from independent experiments. To see this figure in color, go online.

Existing models to compute the kinetic rate constants for the initial reaction of tetramers into ULFs (12) do not consider that the diameter of the ULFs may increase at higher salt concentrations. Thus, in the following, we do not compute absolute kinetic rate constants but instead describe the influence of salt concentration on the reaction speed relative to the reaction at low-salt concentration. We do this by computing the ratio of the scattered light signals, separately for each wavelength, between medium- versus low-salt conditions (Fig. 7 *A* and *B*, *green curves*) and between high- versus low-salt conditions (Fig. 7 *A* and *B*, *orange curves*). These ratios contain information about the lateral and longitudinal assembly kinetics as well as the mass per cross section of the filaments relative to the reaction at low-salt conditions.

To decipher this information, we take advantage of the fact that filaments longer than 1 ULF are highly unlikely to have formed in the first 40 ms of the reaction. Thus, the medium-to-low- and high-to-low-salt ratios, when averaged between 20 and 40 ms after starting polymerization, report differences in the lateral assembly kinetics. We obtain the same average values for the ratios measured at 405 and 594 nm, confirming that longitudinal filament assembly has not yet started. For vimentin concentrations of 0.1 mg/mL and higher, we find on average an approximately 1.3-fold faster assembly under medium- compared with low-salt conditions and a 1.48-fold faster assembly under high- compared with low-salt conditions. For a vimentin concentration of 0.05 mg/mL, however, the lateral assembly is only marginally faster at medium- and high-salt compared with low-salt conditions. Moreover, we find a trend toward faster lateral assembly (relative to low-salt conditions) with increasing vimentin concentration.

We take advantage of the fact that toward the end of the measurement, the filaments have reached a length beyond which the 405 nm scattering signal becomes insensitive to any further growth. The signal ratios between medium-to-low- and high-to-low-salt conditions, averaged between 500 and 600 s, therefore report the filament mass per cross section relative to low-salt conditions. We find a 1.3-fold higher mass per cross section for medium salt, and a 1.6-fold higher mass per cross section for high salt, compared with filaments that have assembled under low-salt conditions. These relative differences are slightly more pronounced for lower vimentin concentrations.

Interpreting the signal ratios for intermediate time points (between 1 and 100 s) is not as straightforward, as the scattering signal is influenced by both lateral and longitudinal assembly kinetics. However, since we measure the filament elongation rate for all conditions (Fig. 6), we can, using Rayleigh-Gans theory, estimate the contribution of the elongation kinetics to the scattered light intensities in the absence of any additional lateral growth (Fig. S4). Accordingly, the medium-to-low-salt ratio of the theoretically predicted scattered light intensities for 405 nm shows a peak at around 30 s after starting the polymerization, with a peak value of 1.15. For the high-to-low ratio, the peak value is 1.2 (Fig. S4 *C*). The measured peak ratios, by contrast, are considerably higher and reach values of 1.5 and 2.1 for the medium-to-low and high-to-low ratios, respectively (Fig. 7 *E*). The difference between the theoretically predicted and measured values arises from the larger mass per cross section of the filaments at medium- and high-salt conditions compared with low salt. This difference translates to filaments that have a 1.35-fold larger mass per cross section (for medium salt) and 1.85-fold larger mass per cross section (for high salt) compared with low-salt conditions.

These mass per cross section estimates from the peak ratios are larger than those that we obtain from the plateau ratios (Fig. 7 *G* and *H*). A likely explanation is that the filaments have undergone a maturation process in the form of shedding of loosely associated tetramers, leading to radial compaction. This interpretation agrees with a scanning EM analysis of filament thickness, where we also find that the filament diameter peaks after 30 s of assembly and then decreases by approximately 30% (Fig. 4 *D*).

## Discussion

IFs represent a supermolecular self-assembly system that forms linear polymers from fibrous, highly charged molecules (23). The basic architectural units in IFs are parallel coiled-coil dimers with a conserved central *α*-helical domain of about 46 nm in length. The basic functional unit for filament assembly is a tetrameric complex assembled from anti-parallel, half-staggered coiled-coil dimers, with the amino-terminal halves of the *α*-helical domain forming a tight interaction. Consequently, this interaction restricts the flexibility of the extended rods significantly (24, 25, 26, 27) such that tetramers appear mostly as straight rods in electron microscopic images (8).

When tetramers are induced to assemble laterally into full-width ULFs, their interaction is topologically restricted. Thus, tetramers laterally bind each other in register, as concluded from the observation that the length of a tetramer is identical to that of an ULF, i.e., ∼60 nm (28). Notably, IFs can be dissociated to tetramers in buffers of low ionic strength, and tetramers will reassemble into filaments when the ionic strength is raised (12). This property of the IF system allows us to kinetically analyze the formation of filaments from tetramers simply by an instantaneous change in salt concentration.

The first quantitative kinetic experiments in IFs were performed using EM and AFM, by measuring the time-dependent elongation of vimentin filaments (29). Data recorded from 5 s to 20 min after initiating assembly revealed a two-phase scenario: over the first 10 s, only ULFs were recognized. After 30 s, elongation products were clearly discriminated from single ULFs, and by 60 s, the mean filament length was 130 nm, corresponding to the longitudinal annealing of three ULFs. Over the following 20 min, mean filament length increased linearly over time. However, finer details of assembly during the first 60 s could not be resolved with this technique.

In a previous study, we employed stopped-flow measurements with a time resolution of 3 ms and recorded the assembly of tetramers into the next higher-order complexes such as octamers, 16-mers, and ULFs during the first 500 ms (12). Tetramers were used up within several tens of milliseconds to form octamers, which in return were quickly consumed to generate 16-mers and ULFs. Filament elongation started as soon as the first ULFs appeared. Although lateral assembly and elongation could not be separately measured in this study, the data clearly refuted the concept that IF formation, like F-actin assembly, starts with a mini-filament acting as a “nucleus” onto which single tetramers add on in a helical fashion. A segmental growth of IFs from ULFs is further supported by the behavior of temperature-sensitive human vimentin variants that, at room temperature, stop assembly at the ULF state but elongate as soon as the temperature is increased to 37°C (9). Moreover, long-term assembly experiments with two vimentin species labeled with Alexa 647 or Alexa 488, respectively, that were assembled separately for 1 h and then added together for 2 days revealed that the resulting IFs were strictly organized into red and green segments. This demonstrated that also long filaments, as obtained 1 h after initiation of assembly, grow further by end-on annealing (30). Furthermore, the absence of yellow segments demonstrated that IFs, once formed under these in vitro conditions, do not disassemble into smaller subunits such as tetramers, octamers, or ULFs and then reassemble into mixed red-green (yellow) segments. Until now, however, the early kinetics of filament elongation immediately following ULF formation was not experimentally accessible.

Here, we describe a novel method to simultaneously but separately measure the lateral and longitudinal assembly of IFs using a dual-wavelength stopped-flow device with a temporal resolution of 3 ms. By observing the static light scattering signal at two distinct wavelengths of 405 and 594 nm, we can distinguish lateral assembly, which increases the signals from both wavelengths by the same factor, from longitudinal assembly, which increases predominantly the signal from the longer wavelength. The ratio of the 594–405 nm signal, after subtracting the water background and normalizing the signals during the first 50 ms to the same value, corresponds to the length of rod-like scattering objects if their width is considerably smaller than the wavelength of the incident light, according to Rayleigh-Gans scattering theory. However, if the lengths of the scattering objects are distributed, the average length can only be computed from the 594/405 nm ratio if the shape of the length distribution is known. From AFM measurements, we find that the filament lengths are approximately log-normal distributed and that the width of the distribution, apart from a constant offset, increases linearly with the geometric mean of the filament length (Eq. 5). In practice, we take advantage of the observation that vimentin filaments elongate with a constant assembly rate and that the 594/405 nm ratio increases according to a monoexponential function (Eq. 6). Hence, the time constant of the exponential function is inversely proportional to the longitudinal assembly rate, according to Eq. 7.

The factor of proportionality in Eq. 7 is subject to several uncertainties. First, we assume that the assembly rates and filament length distribution in the AFM and stopped-flow experiments are similar. AFM measurements require fixation of growing filaments and their attachment to a solid support, which has been argued to be a potential source of error in determining the length and diameter of filaments (31,32). However, we demonstrated, by performing EM and AFM on different substrates, that both methods produce highly consistent results (4,29). Second, we further assume that the filament length distribution depends only on the geometric mean of the filament lengths but not on salt or protein concentration. We have not tested this assumption experimentally, but a recent modeling study confirms its validity (33).

We apply our method to investigate the assembly kinetics of vimentin and its dependence on the ion concentration of the assembly buffer. Our data are in line with earlier stopped-flow and EM studies: the early assembly during the first second is dominated by lateral reaction processes. Between 1 and 10 s, longitudinal assembly starts to dominate, which can be observed by the differences in the scattered light intensities at different wavelengths. Lateral and longitudinal assembly processes continue to coexist for the next 10–30 s. This is followed by a phase from about 30 to 90 s, where the filaments continue to grow in length and—dependent on salt and vimentin concentration—shrink in diameter. This reduction in diameter is seen both by EM measurements (Fig. 4) and by the overshoot in the scattered light intensities at 405 nm (Fig. 5 *B*). The reduction in diameter is facilitated by a dissociation of smaller complexes from the filament core and by intrafilamentous reorganization, which is also referred to as radial compaction (13,22). After several minutes, the lateral compaction process comes to a halt, and longitudinal assembly prevails.

We find a strong salt concentration-dependent increase of both the lateral and longitudinal assembly kinetics. Notably, we find that filaments assembled at higher salt concentrations (100 and 160 mM) exhibit a greater mass per cross section and show excess radial growth during the first 30–90 s of assembly compared with assembly at 50 mM salt. At higher salt concentrations, the kinetics of lateral association is accelerated to an extent that heterogeneous ULFs are formed, containing regular 8 to 12 tetramers that are productive for elongation, together with hyper-aggregated assembly complexes that are unstable. Such heterogeneous hyper-aggregates have previously been observed by scanning TEM when filament assembly was triggered by salt addition (13). By contrast, when filaments were assembled by dialysis, IFs were completely homogeneous, with a constant mass distribution among individual filaments (8). This, together with our observation that the signal overshoot during the first 30–90 s of assembly at a lower salt concentration of 50 mM is largely absent, indicates that an ordered lateral assembly needs a certain time for the precise association of tetramers. Notably, different IF proteins vary considerably in their assembly kinetics: keratin K8/K18 assembles about a hundred times faster than vimentin, and the sequence-related desmin assembles five times faster than vimentin (34). Hence, despite the principally identical structural organization of the monomers and the coiled-coil dimers, and the analogous assembly mechanism of these different IF proteins, their individual amino acid sequences appear to have a strong impact on the kinetics of the longitudinal assembly reaction.

An interesting parallel to IF assembly is the formation of synthetic myosin II filaments, also termed “minifilaments.” Like IF proteins, two myosin II molecules form, via their extended *α*-helical rod domains, a parallel coiled-coil dimer, which is termed a monomer in the myosin field (35). Furthermore, a rapid change of the salt concentration initiates a sequential association of monomers to dimers to tetramers to minifilaments, as shown by EM and stopped-flow methods (36, 37, 38). Most of the assembly occurs within 50 ms and is nearly complete by 1 s (38). Hence, the myosin assembly process closely resembles, both in its sequential steps and their kinetics, the lateral association reaction of vimentin tetramers to ULFs (12). However, myosin minifilaments do not elongate further by end-to-end annealing of minifilaments, in stark contrast to IF assembly.

Our findings are in line with a recent report by Lopez and colleagues, who have demonstrated, using static and dynamic light scattering in combination with mass measurements by scanning TEM, that the ionic strength of the assembly buffer influences both the lateral and the longitudinal kinetics of assembly (32). To avoid problems arising when IF proteins are assembled under near-physiological conditions, such as very fast formation of filaments in combination with the formation of extensive filament bundles (39), they employed a low protein concentration (0.035 mg/mL) and an assembly temperature of 20°C (31,32). In such a regimen, the highly active molecular interactions of IF tetramers and the formation of higher-order assembly products are drastically decelerated compared with assembly at 37°C and higher protein concentrations (12). Within 120 min of assembly, they obtained a mean filament length representing four longitudinally annealed ULFs. For comparison, under the conditions employed in our experiments (protein concentration 0.05–0.4 mg/mL, assembly at 37°C), a median length of four ULFs is reached within 1–4 min (Fig. 6). Lopez et al. also noted that at higher salt concentrations, the mass per cross section of the filaments increased (32). Furthermore, the filament growth rate decreased with increasing filament length *L* according to $L^{-4}$. By contrast, our results obtained at 37°C and at higher protein concentrations demonstrate an approximately constant, length-independent growth rate, giving rise to a nearly linear filament growth over time (20).

We have previously demonstrated that vimentins from different species exhibit a temperature-dependent assembly such that above a certain temperature (usually the body temperature of these organisms), the longitudinal assembly is impaired, and instead, band-like fibrous aggregates appear (8,40). Also at low temperature, the formation of loosely associated filaments is prominent, but elongation is impaired, as demonstrated for amphibian vimentin, which assembles best at 28°C but fails to regularly assemble at 4°C (41). Hence, it is not clear in which temperature range vimentin, of a certain species, is assembling in a regular manner. This question can of course now be tackled with our stopped-flow system.

In vivo, the longitudinal assembly is spatially and kinetically governed by a chaperone system involving motor protein-mediated distribution of ULFs, thereby controlling first the subunit composition of ULFs and second the productive elongation within the cell (42). Notably, a bifurcation of filaments is not observed in vitro or in vivo. An ULF segment harboring, for instance, 12 tetramers could theoretically connect to two filaments with 6 tetramers each. This has, however, never been observed by us in hundreds of EM and AFM micrographs, indicating that, even if such an event would take place, the resulting structure would not be stable.

The salt concentration-dependent speed up of assembly is likely caused by the efficient shielding of ionic charges between the ionic amino acid side chains within and between dimers. Hence, with more monovalent ions being present, equal ionic charges are hindered from repelling each other, and salt bridges may form more readily. As a result, organized interactions leading to productive assembly may proceed more efficiently. This is of great importance for disease mutants of both vimentin and desmin. They have previously been described to leave the regular assembly pathway at distinctly different time points (18,43, 44, 45). Accordingly, we predict that assembly of such mutated IFs requires higher salt concentrations compared with wild-type IFs. Indeed, the desmin mutant R406W-desmin, which causes severe myopathies and cardiomyopathies in humans, was characterized to assemble not much beyond the ULF state when assembly was conducted in 50 mM sodium chloride, a condition where wild-type desmin rapidly forms very long filaments (18). By contrast, when assembly was conducted at 160 mM sodium chloride, R406W-desmin exhibited the same assembly kinetics as wild-type desmin during the first 30 s of assembly. By this time, however, the nascent filaments agglomerated laterally into large aggregates, thereby blocking further longitudinal annealing. As a result, assembly terminated with the accumulation of unproductive non-IF aggregates (17).

## Conclusion

The measurement of static light scattering at two different wavelengths in a stopped-flow device allows for the calculation of the shape factor of the reaction products. This in turn can be used to infer the kinetics of both the lateral and longitudinal assembly of IFs with unprecedented temporal resolution. Moreover, these experiments can now be performed under a wide range of conditions, such as different temperatures and ionic strengths or the presence of chaperones, cross linkers, or reactive oxygen species, to explore the extraordinary functional versatility of the IF system.

## Author contributions

N.M. conceived the method and modified the stopped-flow device for dual-wavelength measurements, N.M., H.H., W.H.G., and B.F. designed the experiments; N.M., H.H., B.F., W.H.G., and L.S. carried out the experiments; L.S., B.F., and N.M. analyzed the data; S.P. performed the simulations; and N.M., H.H., B.F., and L.S. wrote the manuscript.

## Supporting material

Document S1. Figures S1–S4

Document S2. Article plus supporting material
