# Supplementary material for: Dual-wavelength stopped-flow analysis of the lateral and longitudinal assembly kinetics of vimentin
Source: Biophys J. 2022 Sep 13;121(20):3850–61. doi: 10.1016/j.bpj.2022.09.008 (PMC9674981; doi:10.1016/j.bpj.2022.09.008)
Supplement: Document S1. Figures S1–S4 [file mmc1.pdf]

Biophysical Journal, Volume *121*

Supplemental information

Dual-wavelength stopped-flow analysis of the lateral and longitudinal assembly kinetics of vimentin

Lovis Schween, Norbert Mücke, Stéphanie Portet, Wolfgang H. Goldmann, Harald Herrmann, and Ben Fabry

Dual-wavelength stopped-flow analysis of the lateral and longitudinal assembly kinetics of vimentin

Lovis Schween[1,5,a]**, Norbert Mücke**[2,a]**, Stéphanie Portet**[3]**, Wolfgang H. Goldmann**[1,5]**, Harald Herrmann**[4,5]**, and Ben Fabry**[1,5]

[1]Department of Physics, Friedrich-Alexander-Universität Erlangen-Nürnberg, Erlangen, Germany
[2]Division of Chromatin Networks, German Cancer Research Center, Heidelberg, Germany
[3]Department of Mathematics, University of Manitoba, Winnipeg, MB, Canada
[4]Institute of Neuropathology, University Hospital Erlangen, Erlangen, Germany
[5]Muscle Research Center Erlangen (MURCE), Friedrich-Alexander-Universität Erlangen-Nürnberg, Erlangen, Germany
[a]both authors contributed equally

Supplementary Note 1: Figures

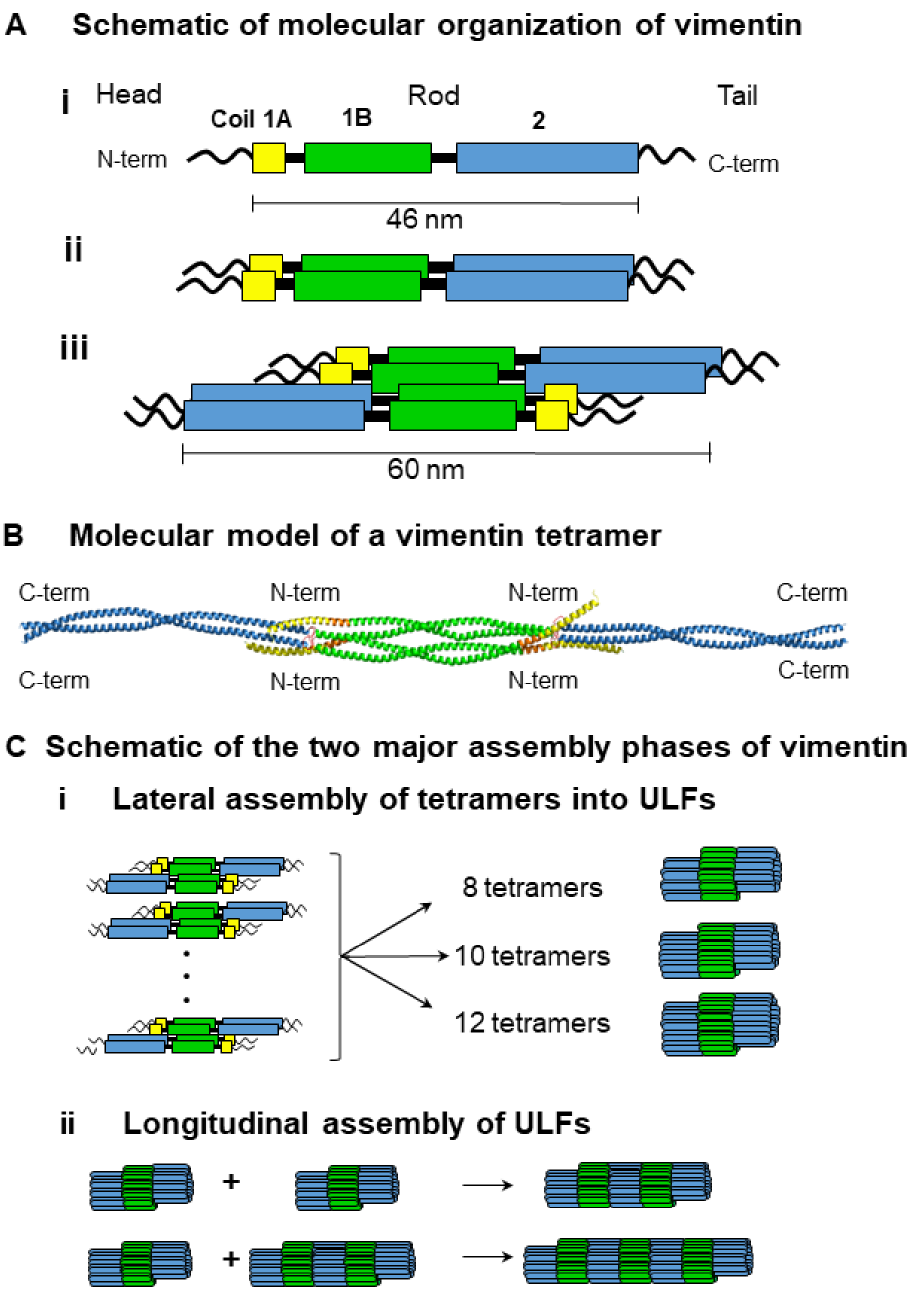

Fig. SI 1. Molecular organization and assembly phases of vimentin. **A)** (i) A vimentin monomer consists of a 46 nm-long extended central "rod" domain, which exhibits three alpha-helical coil segments, termed coil 1A, 1B, and 2. These three alpha-helical segments are connected by so-called linker regions (thick black bars). The colors of the boxes correspond to those of the domains depicted in the atomic model below in (B). The rod domain is flanked by an amino-terminal (N-term) and a carboxy-terminal (C-term) domain, referred to as "head" and "tail", respectively. Both domains are supposed to be unstructured (wavy lines). (ii) Two vimentin monomers form a parallel coiled-coil dimer. (iii) Under low ionic strength, two dimeric complexes assemble in an anti-parallel and half-staggered fashion into 60 nm long tetramers, with the coil 1 segments in direct contact in the center of the tetramer; hence, tetramers are non-polar. (Adapted from (1)). **B)** Molecular model of a vimentin tetramer. The basic functional unit for filament assembly, the tetramer, exhibits an anti-parallel overlap of the coil 1 segments of two dimeric complexes. (Adapted from (2)). **C)** Schematic of the two major assembly phases of vimentin. (i) eight, ten, or twelve tetramers assemble laterally into unit-length filaments (ULFs) (3). (ii) ULFs assemble longitudinally, via end-to-end annealing, into short vimentin filaments, which subsequently further elongate by end-to-end annealing. (Adapted from (4)). Timescale of vimentin assembly under physiological conditions: Lateral assembly predominantly in the first second, co-existence of lateral and longitudinal assembly between 1-30 s, thereafter mostly longitudinal assembly.

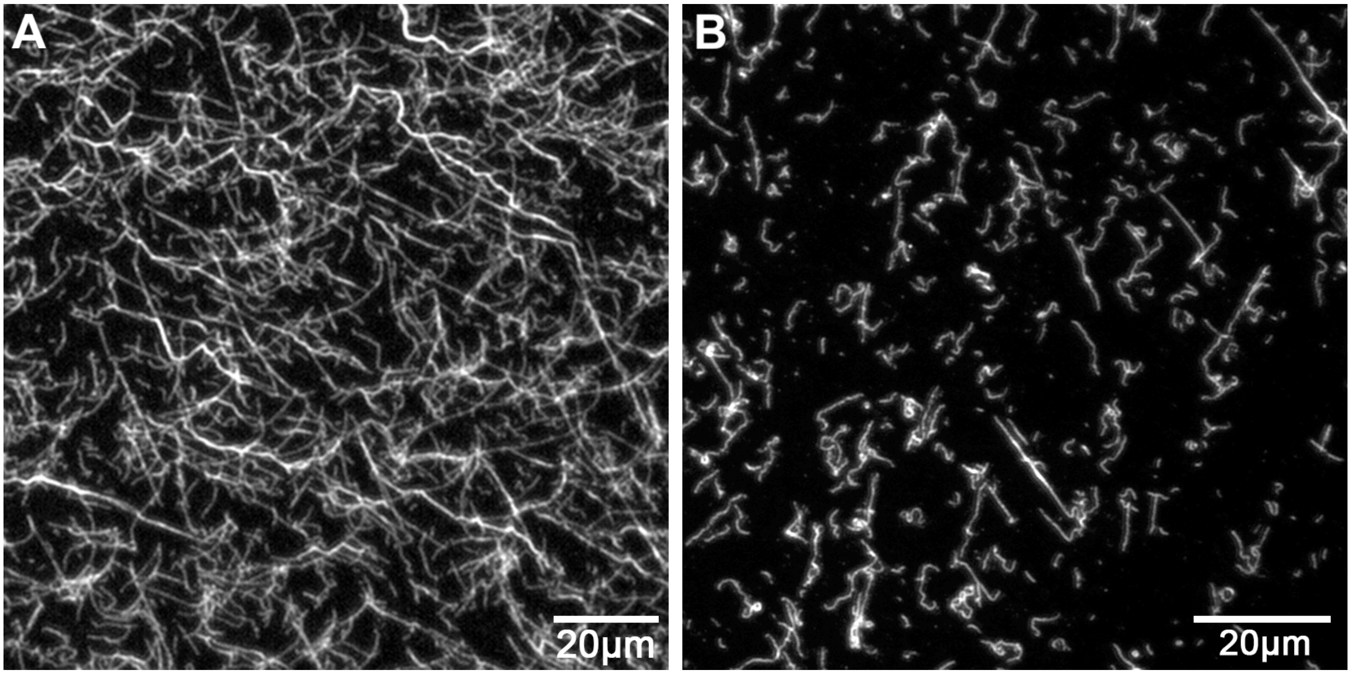

Fig. SI 2. Immunofluorescence microscopic images of vimentin filaments after 60 min of assembly (0.2 mg/ml, 160 mM NaCl). **A)** The filaments form a complex network. **B)** In a 1:50 diluted suspension, single unbranched and highly flexible filaments are visible. Scale bar: 20 μm.

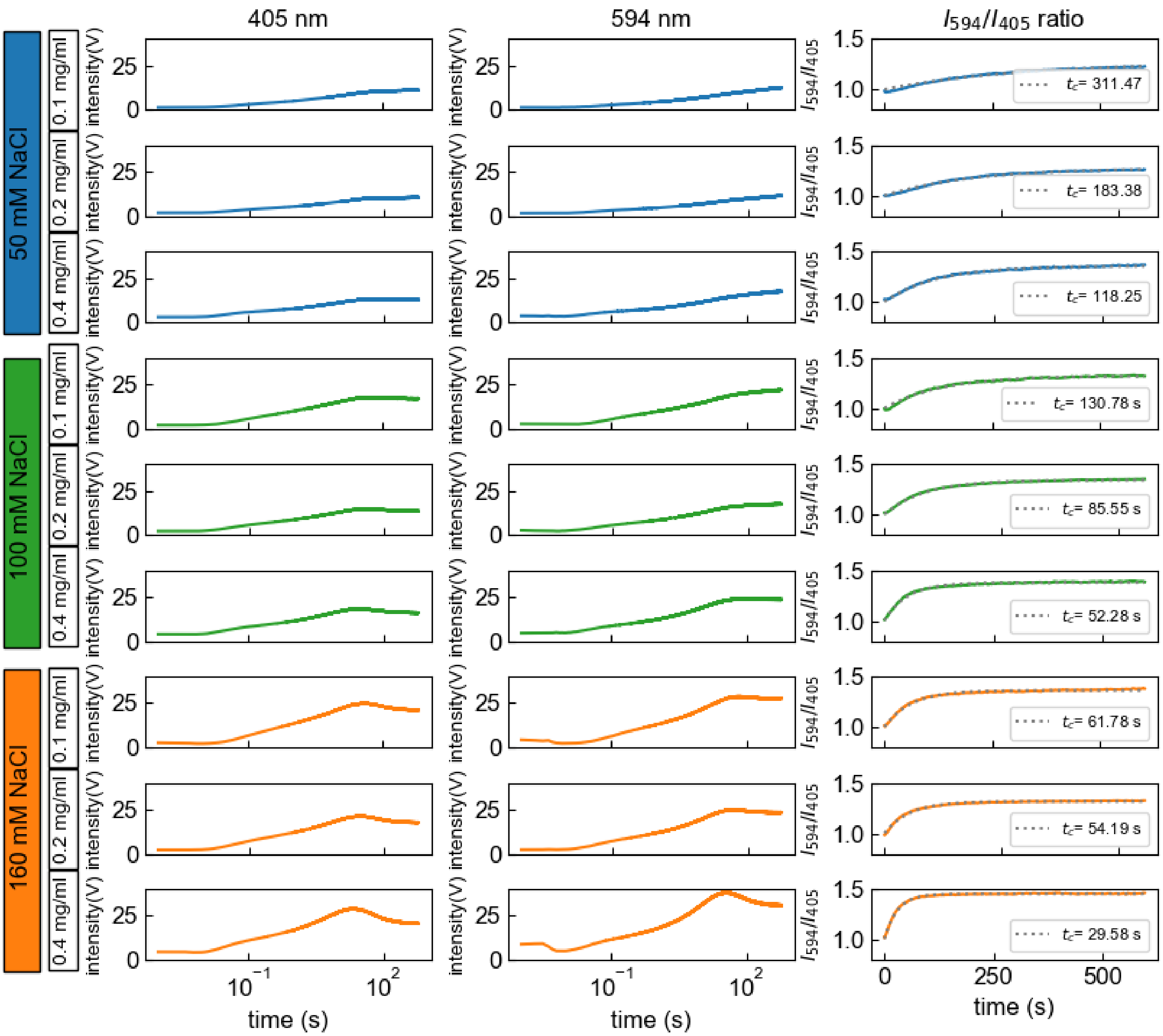

Fig. SI 3. Normalized mean scattered light intensities at 405 nm and 594 nm and their intensity ratio for NaCl concentrations of 50 mM (blue), 100 mM (green) and 160 mM (orange), and for vimentin concentrations of 0.1 mg/ml, 0.2 mg/ml and 0.4 mg/ml. The time constant t_c of the exponential fit of the intensity ratio signal, as displayed in each figure, decrease with increasing vimentin and salt concentration.

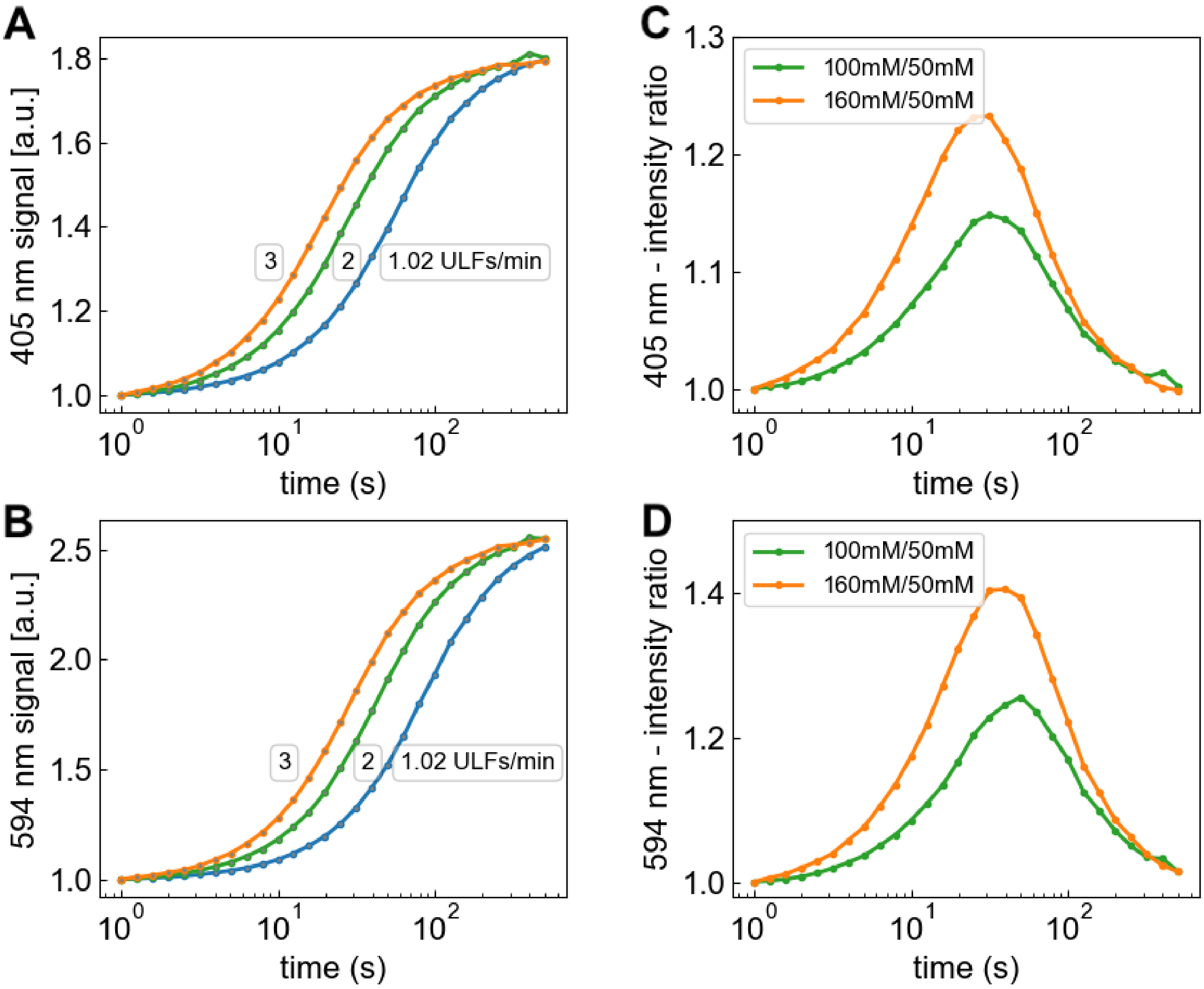

Fig. SI 4. Simulated scattering intensity during longitudinal assembly **(A,B)** for growth rates 1.02 (blue), 2.0 (green) and 3.0 ULFs/min (orange). These growth rates correspond to the assembly of 0.2 mg/ml vimentin in 50 mM (blue), 100 mM (green), and 160 mM (orange) sodium chloride, respectively. The simulated scattered light curves for 100 mM (green) and 160 mM (orange) sodium chloride are divided by the scattered light curve of 50 mM (blue) NaCl **(C,D)**. The different assembly speeds for each salt condition result in a peak in the 100 mM / 50 mM (green) and the 160 mM / 50 mM (orange) signal ratios between 30 s and 90 s, but beyond 500 s, the ratios return to unity as the filaments under all conditions have elongated to a length of >10 ULFs beyond which the scatter signal shows little or no further increase. The measured signal ratios (Fig. 7), by contrast, do not return to unity, indicating that the mass per cross-section of the filaments increases with increasing salt concentrations.

References

1. Denz, M., M. Marschall, H. Herrmann, and S. Köster, 2021. Ion type and valency differentially drive vimentin tetramers into intermediate filaments or higher order assemblies. *Soft Matter* 17:870–878.
2. Chernyatina, A., S. Nicolet, U. Aebi, H. Herrmann, and S. Strelkov, 2012. Atomic structure of the vimentin central α-helical domain and its implications for intermediate filament assembly. *Proceedings of the National Academy of Sciences* 109:13620–13625.
3. Herrmann, H., M. Häner, M. Brettel, S. Müller, K. Goldie, B. Fedtke, A. Lustig, W. Franke, and U. Aebi, 1996. Structure and Assembly Properties of the Intermediate Filament Protein Vimentin: The Role of its Head, Rod and Tail Domain. *J. Mol. Biol.* 264:933–953.
4. Lopez, C., O. Saldanha, K. Huber, and S. Köster, 2016. Lateral association and elongation of vimentin intermediate filament proteins: A time-resolved light-scattering study. *Proceedings of the National Academy of Sciences* 113:11152–11157.
